# Source Apportionment and Influencing Factor Analysis of Residential Indoor PM_2.5_ in Beijing

**DOI:** 10.3390/ijerph15040686

**Published:** 2018-04-05

**Authors:** Yibing Yang, Liu Liu, Chunyu Xu, Na Li, Zhe Liu, Qin Wang, Dongqun Xu

**Affiliations:** National Institute of Environmental Health, Chinese Center for Disease Control and Prevention, Beijing 100021, China; yangyibing32@163.com (Y.Y.); liuliu@cycdpc.org (L.L.); xuchunyu@nieh.chinacdc.cn (C.X.); lina@nieh.chinacdc.cn (N.L.); liuzhe@nieh.chinacdc.cn (Z.L.); wangqin@nieh.chinacdc.cn (Q.W.)

**Keywords:** indoor PM_2.5_, residential houses, chemical elements, source apportionment, influencing factor analysis

## Abstract

In order to identify the sources of indoor PM_2.5_ and to check which factors influence the concentration of indoor PM_2.5_ and chemical elements, indoor concentrations of PM_2.5_ and its related elements in residential houses in Beijing were explored. Indoor and outdoor PM_2.5_ samples that were monitored continuously for one week were collected. Indoor and outdoor concentrations of PM_2.5_ and 15 elements (Al, As, Ca, Cd, Cu, Fe, K, Mg, Mn, Na, Pb, Se, Tl, V, Zn) were calculated and compared. The median indoor concentration of PM_2.5_ was 57.64 μg/m^3^. For elements in indoor PM_2.5_, Cd and As may be sensitive to indoor smoking, Zn, Ca and Al may be related to indoor sources other than smoking, Pb, V and Se may mainly come from outdoor. Five factors were extracted for indoor PM_2.5_ by factor analysis, explained 76.8% of total variance, outdoor sources contributed more than indoor sources. Multiple linear regression analysis for indoor PM_2.5_, Cd and Pb was performed. Indoor PM_2.5_ was influenced by factors including outdoor PM_2.5_, smoking during sampling, outdoor temperature and time of air conditioner use. Indoor Cd was affected by factors including smoking during sampling, outdoor Cd and building age. Indoor Pb concentration was associated with factors including outdoor Pb and time of window open per day, building age and RH. In conclusion, indoor PM_2.5_ mainly comes from outdoor sources, and the contributions of indoor sources also cannot be ignored. Factors associated indoor and outdoor air exchange can influence the concentrations of indoor PM_2.5_ and its constituents.

## 1. Introduction

The majority of adults spend more than 80% of the day in a variety of indoor environments, mainly in their houses [1,2,3]. The contribution of indoor air is very important for accurate estimation of individual air pollution exposure. A vast number of epidemiological studies have shown that both short and long-term exposure to fine particulate matter (PM_2.5_) are harmful to the human respiratory and cardiovascular systems [4,5,6,7,8,9,10]. However, the PM_2.5_ exposure assessment in most of these studies is usually based on ambient PM_2.5_ data, regardless of the difference between indoor and outdoor PM_2.5_ concentration. And failure to account for the contribution of indoor PM_2.5_ concentration may lead to exposure misclassifications, which have become source of measurement bias in the PM_2.5_ epidemiologic studies linking health effects to PM_2.5_ exposure [11,12,13,14]. 

Indoor PM_2.5_ comes from both outdoor and indoor sources. Outdoor PM_2.5_ typically comes from sources such as fossil fuels combustion, motor vehicle exhaust, industrial emissions, soil and dust, and particles from these sources can penetrate into indoor through building gap, door, window and mechanical ventilation [12,15,16,17,18]. A survey conducted in Beijing showed that 54–63% of indoor PM_2.5_ originated from the outdoors with all windows closed [15]. Indoor sources such as smoking, cooking and indoor activities, also can generate particles to increase indoor PM_2.5_ concentration, those sources have been recognized as another important contributor of indoor PM_2.5_ [19,20,21,22,23]. 

The source apportionment of outdoor PM_2.5_ have been explored in many studies across the world [16,17,18,24,25,26,27,28], but information about source apportionment of indoor PM_2.5_, especially for residential houses, is relatively scarce. Chemical elements, including metal and metalloid elements, are common constituents of PM_2.5_ in urban cities, and the use of elements as tracers to identify PM_2.5_ from different sources has been applied in many studies [16,25,29,30,31,32,33,34,35,36]. Chemical elements in outdoor PM_2.5_ can be divided into several tracer groups for different sources, such as, Al, Mg and Ca which are usually used as trace elements for soil dust, V and Se are usually used as trace elements for fossil fuel combustion; Pb and Cu are usually used as trace elements of motor vehicle exhaust [16,29,30,31,32,33,34,35,36]. Indoor sources can also provide chemical elements for PM_2.5_. Elements such as Cd, As, Cu, Ni, Hg, Mn and Al have been detected in cigarette smoke [37,38,39,40], and Zn, Al, Mg and Ca can be found in cooking fumes [41,42,43]. 

Metal and metalloid elements attached to particles can enter the body through breathing. Previous studies have also addressed that those elements in PM_2.5_ are linked to diseases such as stroke, pneumonia and asthma [44,45,46,47]. Besides, the toxicity of elements on human health has been reported in many studies [48,49,50,51,52,53], especially for Cd and Pb, whereby exposure to low concentrations of Cd was associated with renal toxicity, osteoporosis and bone fractures [49,50,51], and low Pb exposure could lead to neurotoxicity and immune system damage [52,53], so metal and metalloid elements have been suspected to play an important role in the health effects of PM_2.5_ [16,47,54,55]. Therefore, understanding concentrations of metal and metalloid elements is essential not only for source apportionment of indoor PM_2.5_, but also for controlling those sources to reduce the health hazards.

Additionally, there are factors can affect the exchange of indoor and outdoor air, such as window opening behaviors, building characteristics, air conditioner use, and outdoor meteorological variables, etc., and these factors may also have an impact on the concentration of indoor PM_2.5_ and its constituents [12,13,22,56,57,58,59,60,61,62,63,64].

Concentrations of indoor PM_2.5_ and related chemical elements in residential house have been explored in several individual cities, such as in Kocaeli City [65], Saint Paul [55], Antwerp [66] and other cities [67,68,69], but the levels and sources of PM_2.5_ pollution, meteorological conditions, human behaviors and building characteristics are not always the same in different cities. In Beijing, ambient PM_2.5_ pollution has been serious in recent years, especially in autumn and winter [18,30,70]. Ambient PM_2.5_ pollution in Beijing is dominated by vehicle emissions [71,72], but fossil fuels combustion from heating activities has become another important contributor to ambient PM_2.5_ during the heating season [30,31,73]. Several studies have investigated indoor PM_2.5_ concentrations and their relationship with outdoor PM_2.5_ in residential houses in Beijing [58,59,60,61,62,63], but the information about concentrations of metal and metalloid elements in indoor PM_2.5_ is quite rare [74,75,76]. In this study, indoor and outdoor PM_2.5_ of residential houses were continuously monitored for one week during the non-heating season (NHS) and heating season (HS) in Beijing. The concentrations of PM_2.5_ and 15 kinds of related elements (Al, As, Ca, Cd, Cu, Fe, K, Mg, Mn, Na, Pb, Se, Tl, V, Zn) were calculated, indoor and outdoor concentrations of PM_2.5_ and its constituents were compared. The possible sources of indoor PM_2.5_ and potential influencing factors of indoor PM_2.5_ and its constituents’ concentrations were analyzed.

## 2. Methods

### 2.1. Participant Recruitment

The participants were recruited by telephone interview to determine the building type and floor of the houses. Since the majority of citizens in Beijing live in apartment buildings, and a certain percentage of citizens live in villas and courtyards, all three types of building were surveyed. Apartment buildings were divided into three groups based on the building height: 1–3 floors, 4–9 floors and 10 floors or above. Stratified sampling was used to select houses based on their building type and floor group, and there were at least five houses in each type of building and each group of apartments.

### 2.2. Indoor and Outdoor PM_2.5_ Sampling

The indoor and outdoor sampling was conducted in two seasons: August 2013–October 2013 was defined as the non-heating season, and November 2013–March 2014 was defined as the heating season. Each house was sampled for a continuous seven-day during the non-heating season and heating season. Indoor samplers were placed in the living room (if the living room was not available, then placed in a bedroom). The intake port of the outdoor sampler was extended at least 1 meter outside the available windows that were hardly used, and away from the outlet of the air conditioner (AC) or extractor hood. The PM_2.5_ samples of the indoor and outdoor areas were collected at the same time by a PCXR8-Universal Sampling Pump (SKC, Eighty Four, PA, USA). The sampling target flow rate was 0.5 L/min. Samples with flow rates more than 20% different from the target rate were considered invalid. The indoor and outdoor PM_2.5_ was collected on 37 mm Teflon PTFE filter and their mass concentrations were measured by the gravimetric method. Daily outdoor atmospheric pressure, temperature, relative humidity (RH) and wind speed during sampling periods were obtained from the National Meteorological Information Center (Beijing, China). More information about indoor/outdoor sampling had been previously described [58]. 

### 2.3. Questionnaires and Recoding Sheets

Questionnaires and recoding sheets were investigated during the sampling to obtain the information of potential influencing variables of indoor PM_2.5_. A baseline questionnaire was completed by each house at the beginning of indoor/outdoor sampling in non-heating season, to collect variables of building characteristics and general inhabitant behaviors. At the end of each sampling in two seasons, a sampling questionnaire was completed to observe the consistency and diversity of resident’s behavior during sampling period, compared to information from baseline questionnaire. The main information of these two questionnaires was shown in Table S1 in the Supplementary Materials. The residents also recorded information on cooking, window opening and air conditioner (AC) use every day on a recording sheet.

### 2.4. Determination of PM_2.5_ and Chemical Elements

The mass concentrations of PM_2.5_ were measured with an analytical balance (XP6, Mettler Toledo, Zurich, Switzerland, verification scale value of 0.001 mg). Before and after sampling, the filters were pre-conditioned for a minimum of 24 h at a constant temperature of 25 °C (±1 °C) and constant RH of 50% ± 5% before weighing.

The PM_2.5_ mass concentrations of indoor and outdoor areas (ρ, μg/m^3^) were calculated with the following equation:

(1) $$\rho =\frac{w_{2}-w_{1}}{V}\times 1000$$

The weight mass of the filter before sampling (w_1_, mg) and the weight mass of the filter after sampling (w_2_, mg) were measured with the same analytical balance mentioned above. The sampling volume was calculated by the recorded sampling cumulative time and the flow rate of the pump and then converted into the standard state (V, m^3^).

After PM_2.5_ weight analysis was completed, the elements concentrations of each sample were analyzed by inductively coupled plasma mass spectrometry analysis method (ICP-MS, NexION 300D, PerkinElmer, Waltham, MA, USA). Half of each Teflon filter were extracted with 7 mL of nitric acid (HNO_3_, 69%), 0.5 mL of hydrochloric acid (HCL, 36–38%) and 0.5 mL of hydrofluoric acid (HF, 47–51%). The purity of HNO_3_, HF and HCL was guaranteed reagent (GR). The digested solutions were allowed to pre-digest for 2 h in room temperature before loading into the microwave digestion system (MARs5, CEM, Matthews, NC, USA). A stepped microwave program was set: 5 min at 80 °C, followed by 5 min at 120 °C, 5 min at 160 °C, 20 min at 200 °C, and then cooled to room temperature. After the above stepped digestion process, the digestion tanks were opened and the tube wall were rinsed with ultrapure water, then the tank were set into acid-driven processor (BHW-09C, Botonyc Company, Shanghai, China) at 160 °C to clean up the acid to about 1 mL. At last, the extracts were diluted to 10 mL with ultrapure water for ICP-MS analysis. A summary of the operation conditions of the ICP-MS used in this study is shown in Table S2 in the Supplementary Materials. The detection limit of elements in this study was characterized by background equivalent concentration (BEC) value of 10 μg/L standard solution in standard mode (STD), summary of detection limits for chemical elements was shown in Table S3 in Supplementary Materials, and one-half of the detection limit of elements was used for the treatment of values below the detection limit.

### 2.5. Statistical Analysis

Distribution of household characteristics and meteorological variables, indoor and outdoor concentrations of PM_2.5_ as well as related elements was analyzed by descriptive statistical analysis. Since the concentrations of PM_2.5_ and its constituents were not normal distribution tested by Kolmogorov-Smirnov test, comparison of indoor and outdoor concentrations of PM_2.5_ and its constituents was performed by Wilcoxon signed-rank test. Mann-Whitney U test was used to compare the concentrations of PM_2.5_ and its constituents in different groups. Indoor/outdoor correlation coefficient of PM_2.5_ and its constituents was evaluated by spearman correlation analysis. A varimax rotated factor analysis (FA) was performed base on indoor elements data to identify sources of indoor PM_2.5_ in all residential houses. First, based on the concentrations data of elements in indoor PM_2.5_, correlation matrix for each element was obtained. Then, by solving the characteristic equation of the correlation matrix, eigenvalues of factors and factor loading matrix was calculated. At last, the varimax rotated method was used to maximize the sum of the variance of each factor by orthogonally rotating the factor loading matrix, which can contribute to clarify the professional meaning of each factor. The eigenvalue was set to 1.0 as a threshold to limit the number of extracted factors. 

The relationship between indoor concentrations of PM_2.5_ and its constituents and their potential influencing variables were examined by multivariate linear regressions analysis. Indoor concentrations of PM_2.5_ and selected elements were log-transformed to achieve a normal distribution (confirmed by the Kolmogorov-Smirnov test), then they were set as dependent variables, potential influencing variables were set as independent variables. A stepwise variable selection procedure was used for the multivariate linear regression, with an entry level of 0.10 and an exclusion level of 0.05. Season-specific analyses were constructed under the assumption that relationship between indoor PM_2.5_ and potential variables would vary in two seasons. All statistical analyses were performed in the SPSS 22.0 software (SPSS Inc., Chicago, IL, USA), and the significance level was 0.05.

## 3. Results 

### 3.1. Summary of Sampled Houses

A total of 52 houses in NHS and 55 houses in HS were sampled. After excluding the failed samples caused by filter damage and invalid flow rates, 47 houses in NHS and 47 houses in HS were left for further analysis. There were 28 apartments, five villas and 14 courtyards in NHS, and 26, five, 16 in HS, respectively. The descriptive household characteristics of these houses were provided in Table 1. Among the 47 houses in HS, 37 houses were successfully sampled in both seasons.

### 3.2. Distribution of Potential Influencing Variables for Indoor PM_2.5_

More than half of the houses were more than 20 years old. Most houses used liquefied petroleum gas and natural gas for cooking, and hoods were also used during cooking. The ratio of houses with indoor smoking was 10/47 in both seasons. No house used air cleaner and fresh air system during sampling. In HS, most houses used centralized heating, and no house used AC. In NHS, nine houses used an AC, and the average cumulative time of AC use during sampling was 6.8 h (range from 0.0–50.2 h). The median time of window opening per day was 14 h in NHS, but only 0.5 h in HS, respectively, 19 houses did not open window during sampling in HS. Outdoor temperature, average wind speed and atmospheric pressure were different in two seasons. The mean ± standard deviation of average outdoor temperature, average wind speed and atmospheric pressure in the NHS were 20.34 ± 6.18 °C, 8.05 ± 1.15 m/s and 101.24 ± 0.82 kPa, respectively, whereas they were 0.14 ± 2.58 °C, 11.80 ± 2.81 m/s and 102.64 ± 0.43 kPa, respectively, in the HS. The RH had no difference between two seasons.

### 3.3. Residential Indoor and Outdoor Concentration of PM_2.5_ and Elements

Table 2 provides descriptive data for PM_2.5_ and element concentrations of the indoor and outdoor areas. The median (Interquartile Range, IQR) of indoor and outdoor PM_2.5_ concentrations for all houses were 57.64(27.51) μg/m^3^ and 72.49(43.53) μg/m^3^. The median indoor concentration of 15 elements ranged from 0.53 ng/m^3^ to 930.24 ng/m^3^. For As and Se, indoor concentrations of about one-third houses were below the detection limits. The median (IQR) concentration of indoor PM_2.5_ was 55.44(24.12) μg/m^3^ in NHS and 58.23(13.28) μg/m^3^ in HS, and indoor PM_2.5_ were significantly lower than outdoor in both seasons. Outdoor concentrations of all 15 elements were higher than indoor in HS, but for Fe, As and Ca, indoor and outdoor concentrations were not different in NHS.

Spearman correlation analysis was used to evaluate the correlation of indoor and outdoor, results showed that indoor and outdoor PM_2.5_ concentrations were significantly correlated (*p* < 0.01) in both seasons, the correlation coefficient (*r*) was 0.56 in NHS and 0.44 in HS. Indoor Fe and Ca were not correlated with outdoor in both seasons, and indoor Al, Mn, Cu and Mg only correlated with outdoor in NHS. Indoor Pb concentration was highly correlated with outdoor, *r* = 0.73 in NHS and 0.85 in HS.

### 3.4. Elements in Indoor PM_2.5_ that Were Sensitive to Indoor Smoking

Houses with indoor smoking during the sampling were defined as smoking houses, and houses without indoor smoking were defined as non-smoking houses. Concentrations of PM_2.5_ and its constituents for smoking houses and non-smoking houses were compared in Table 3. For smoking and non-smoking houses, median indoor PM_2.5_ concentrations were 66.92 μg/m^3^ and 53.51 μg/m^3^ in NHS, 129.34 μg/m^3^ and 54.60 μg/m^3^ in HS, respectively. After comparing the indoor concentrations of PM_2.5_ and its constituents between smoking houses and non-smoking houses, indoor Cd was higher in smoking houses than in non-smoking houses in both seasons, the difference for indoor PM_2.5_ were only seen in HS. Indoor concentrations of PM_2.5_ and its constituents for smoking houses were also compared with their outdoor concentrations. Results showed that indoor and outdoor PM_2.5_ were no longer different in both seasons, indoor As in NHS and Cd in both seasons were higher than outside. Therefore, Cd and As in indoor PM_2.5_ may be the sensitive elements of indoor smoking.

### 3.5. Elements in Indoor PM_2.5_ that Were Related to Indoor Sources other than Smoking

In HS, residents of 19 houses did not open any windows during sampling, five of those 19 houses had indoor smoking. Indoor and outdoor concentrations of PM_2.5_ and its constituents for these houses were compared in Table 4, to find the elements in indoor PM_2.5_ related to indoor sources other than smoking. For the 19 houses, indoor concentrations of PM_2.5_, Al, Zn, As, Cd, Na and Ca were not different from outdoor, while other elements in indoor PM_2.5_ were significantly lower than outdoor. After excluding the five houses with smoking, the median indoor concentration of PM_2.5_ decreased from 69.53 μg/m^3^ to 61.77 μg/m^3^, indoor PM_2.5_, Cd, As and Na became significantly lower than outdoor in these 14 houses, however, the indoor concentrations of Zn, Al and Ca were still not different from outdoor. In Table 3, concentrations of all elements in indoor PM_2.5_, including Zn, Al and Ca, were significantly lower than outdoor for 37 non-smoking houses in HS, so the result in Table 4 that indoor and outdoor concentrations of Zn, Al and Ca were not different in these 14 houses may be due to the contribution of indoor sources other than indoor smoking. 

Among the 14 houses, 13 houses had indoor cooking, and Zn was reported to be one of the most abundant metals in the cooking fumes, therefore the result of high indoor concentration of Zn may be due to indoor cooking. Al and Ca can be found in cooking fumes from previous studies, but they may also come from re-suspended indoor dust caused by human activities, so the high indoor concentrations of Al and Ca may be due to the indoor cooking and re-suspended dust.

### 3.6. Elements in Indoor PM_2.5_ that Mainly Came from Outdoor

Among all houses, 37 houses were successful sampled in both seasons. A Wilcoxon signed-rank test was used to compare the concentrations of PM_2.5_ and its constituents between two seasons for those houses, and the results are presented in Table 5. Outdoor PM_2.5_ concentrations were much higher in HS, while indoor PM_2.5_ concentrations were not different in the two seasons. Outdoor concentrations of Pb, V, Se and Tl did not differ between the two seasons, but indoor Pb, V, Se and Tl were lower in HS, except that Pb was not significant. From the results in Table 3, indoor concentrations of Pb, Se in HS and V in NHS were significantly lower than outdoors for smoking houses, suggested that Pb, V, Se was not sensitive to indoor smoking. And from the result in Table 4, Pb, V and Se was also not related to other indoor sources. So, Pb, V and Se in indoor PM_2.5_ may mainly come from ambient air.

### 3.7. Factor Analysis of Indoor PM_2.5_

Results of factor analysis were showed in Table 6. Factors loadings equal to or greater than 0.4 are presented in Table 6, factors loadings smaller than 0.40 were omitted from the table, the purpose of which was to facilitate the identification of indoor PM_2.5_ sources. The possible source types were identified based on previous published studies and above analysis. Five factors were extracted from indoor elements data in PM_2.5_, which explained about 76.8% of the total variance, with the first factor (F1) accounting for 21.6%, the second (F2) 21.3%, the third (F3) 13.8%, the fourth (F4) 11.4%, and the fifth (F5) 8.8%. F1 showed high loadings for Fe, V, Mn, Cd and Cu, and these elements all related to products of fossil fuels combustion, so F1 was identified as combustion factor. F2 contained high loadings for Pb, Se, Tl and K, and moderately loadings for Cu and Mn. Elements such as Pb, Cu and Mn can be generated from motor vehicles, but Se, Pb, Tl and K related to products of fossil fuel combustion, so F2 might be factor that combined motor vehicles and combustion. F3 correlated with Zn, Ca and Mg, Zn and Ca may be related to indoor cooking from above results, therefore F3 was identified as indoor cooking factor. F4 involved with Al, Na and Mg, and these compounds have been associated with dust and soil. F5 correlated with As and Cd, these two elements may be sensitive to indoor smoking as previously mentioned, so F5 was identified as an indoor smoking factor. Besides, the factor scores of F5 in smoking houses were significantly higher than in no-smoking houses (*p* < 0.01), which in turn confirmed the identification of F5.

### 3.8. Influencing Factor Analysis of Indoor PM_2.5_ and Selected Elements 

Multiple linear regression analysis was performed for log-transformed indoor PM_2.5_, Cd and Pb to identify their influencing factors. Cd was chosen to represent smoking activity because it was consistently sensitive to indoor smoking from the above analysis. Pb was chosen to represent outdoor sources because indoor Pb was not sensitive to indoor smoking and cooking, and it was highly correlated with outdoor Pb in both seasons. 

The results of multiple linear regression analysis were shown in Table 7. Coefficient of determination (*R*^2^) reflects the percentage variance of indoor PM_2.5_ and its constituents that the influencing variables could explain. In the NHS, indoor PM_2.5_ concentration was mainly influenced by outdoor PM_2.5_ concentration, smoking during sampling and outdoor temperature and time of air conditioner use during sampling in NHS (*R*^2^ = 0.60), it was influenced by outdoor PM_2.5_ and smoking during sampling in HS (*R*^2^ = 0.46). Indoor Cd concentration was mainly influenced by smoking during sampling and outdoor Cd concentration in NHS (*R*^2^ = 0.51), it was influenced by smoking during sampling, outdoor Cd concentration and building age in HS (*R*^2^ = 0.63). Indoor Pb concentration was mainly influenced by outdoor Pb concentration and time of window open per day in the NHS (*R*^2^ = 0.63), it was influenced by outdoor Pb concentration, building age and RH in HS (*R*^2^ = 0.74). 

The results of partial regression coefficient (β) for independent variables in Table 7 indicated that high outdoor concentration of PM_2.5_, Cd and Pb can increase their indoor concentration in both seasons. Smoking during the sampling period could lead to high indoor PM_2.5_ and Cd concentrations in both seasons. In HS, indoor concentrations of Cd and Pb may be higher for older houses. In NHS, more hours of window opening may come with higher indoor Pb concentrations, and less hours of air conditioner use may result in higher indoor PM_2.5_ concentration. For meteorological variables, high outdoor temperature may increase indoor PM_2.5_ concentration in NHS, but high RH may decrease indoor Pb in HS.

## 4. Discussion 

Outdoor PM_2.5_ concentration in heating season was much higher than in non-heating season in this study, a seasonal difference that has also been reported in several other studies conducted in Beijing [30,53,77,78], but not consistent with the study conducted in Beijing by Han [60] which may due to the short sampling periods and the frequent and rapid transition between severe pollution events and clean days. Indoor PM_2.5_ were significantly lower than outdoor in both seasons from Table 2, similar results showed in other studies [59,60,61], but indoor PM_2.5_ concentration was not different between two seasons from Table 5, which may mainly due to the low indoor and outdoor exchange in heating season and the contributions of indoor sources. The median concentration of indoor PM_2.5_ was 57.64 μg/m^3^. Median concentrations of elements in indoor PM_2.5_ ranged from 0.53 ng/m^3^ to 930.24 ng/m^3^, in descending order, K > Fe > Na > Al > Ca > Zn > Mg > Pb > Mn > Cu > As > Se > Cd > V > Tl. Elements such as K, Fe, Na, Al and Ca were abundant and widespread in the natural environment, their concentration was generally higher, but the contents of elements such as V, As and Se in PM_2.5_ were relative low, even below the detection limits. 

Five factors with eigenvalue greater than 1.0 were extracted from the factor analysis based on the elements data in Table 6; they could explain about 76.8% of the total variance of indoor PM_2.5_. Since Pb, V and Se in indoor PM_2.5_ were assumed mainly to come from outdoors from the above analysis, and these three elements only showed high loading in F1 and F2, so F1 and F2 were recognized as outdoor factors, and their contribution to the variance of indoor PM_2.5_ was 42.9%. Elements like V, Fe, Cd, Mn, Se, Cu, Pb, Tl and K were detected from products of fossil fuel combustion in previous studies [16,29,30,31,35,36,55]. Combustion of fossil fuels was reported as the main anthropogenic V emission [16,35,55], but V only showed high loading of F1 in this study, and F1 also had high loading for Fe, Mn, Cd, therefore F1 was preliminarily identified as a combustion factor. Vehicle emissions have been identified as an important source for ambient PM_2.5_ pollution in Beijing [30,71,72], especially in the non-heating season, and both gasoline and diesel oil for fuels of motor vehicles originate from fossil fuels (petroleum), so the elements from motor vehicle emissions might be similar to those from fossil fuel combustion. Besides, previous studies also suggested that motor vehicle exhaust may contain Pb and Cu and motor vehicle fuel additives may contain Pb and Mn [16,29,30,36]. Pb, Tl and Se only showed high loadings in F2, so F2 might be a factor that combined motor vehicles and combustion.

F3 and F5 were recognized as indoor source (cooking and smoking) factors, where their contribution was 20.2%. Chemical elements in cooking fumes has been analyzed in several studies, and contents of Al, Zn, Mg and Ca were relatively high in PM_2.5_ samples from different kitchens [41,42,43], and Zn was considered to be one of the most abundant metals in cooking fumes [41,43]. In this study, data of 14 houses without smoking and window opening in heating season were analyzed in Table 4, and found that Zn and Ca may be related to indoor cooking. F3 shows high loading for Zn and Ca, and a moderate loading for Mg, so it was identified as an indoor cooking factor. Chemical elements in cigarette smoking also have been analyzed in many studies [37,38,39,40], Cd and As were detected in a majority of these studies. Of course, there are other elements released from cigarette smoking such as Al, Mn, Cu, etc. [37,38,39], but in this study, data of smoking and non-smoking houses was compared in Table 3, it was found that only Cd and As were sensitive to indoor smoking, especially Cd. A likely explanation is that for houses without smoking, the content of indoor Cd and As was relatively low and other elements like Al and Mn were much higher, proved by the above order of elements concentrations. Once indoor smoking occurred, the contribution of smoking to Cd and As can be observed, but less noticeable for Al and Mn. In addition, a comparative analysis was performed for F5, the factor score of F5 in smoking and non-smoking houses were compared, houses with indoor smoking had higher scores, so it is reasonable to identify F5 as an indoor smoking factor. A study conducted in Kocaeli City [65] also performed factor analysis for indoor PM_2.5_, where cooking factors and smoking factors contributed 9.7% and 10.6% of the variances, close to 11.4% and 8.8% in this study.

F4 was identified as a dust and soil factor, mainly because it had a high loading of Al, Na, Mg, and these three elements are most often found in soil and dust, which may originate from earth and soil in the ambient environment, or from suspended dust of floors and construction [26,29,30,33,49,65]. 

Outdoor PM_2.5_ is an important source of residential indoor PM_2.5_ [12,13,15,64], and it was significantly correlated with indoor PM_2.5_ concentrations in both seasons from the results of Spearman correlation analysis, and similar results also can be found in other studies [20,59,60,61,62,63]. Multiple linear regression analysis in Table 7 showed that with the increase of outdoor concentration, indoor PM_2.5_ concentration also increased in both seasons. Because indoor and outdoor concentrations of Pb were highly correlated in both seasons and indoor Pb was not sensitive to indoor smoking and cooking, it was selected to represent outdoor sources for multiple linear regression. Results showed that indoor Pb concentration only significantly correlated with variables including outdoor Pb concentration, time of window open per day, building age and RH. And standard regression coefficient (β’) of outdoor Pb concentration was much higher than other variables, indicating that indoor Pb in PM_2.5_ did mainly come from outdoors in this study.

Indoor smoking was identified as an indoor PM_2.5_ source, which can cause a sharp short-term increase in PM_2.5_ concentrations [12,61,65]. In the non-heating season, indoor PM_2.5_ concentrations in smoking houses was only slightly higher than in non-smoking houses, but in heating season the concentrations of indoor PM_2.5_ in smoking houses was more than twice as high as in non-smoking houses from Table 3. Results of multiple linear regression analysis also demonstrated that indoor smoking was an important influencing factor of indoor PM_2.5_ in both seasons. In the comparative analysis from Table 3, Cd was the only element that was consistently sensitive to indoor smoking in both seasons, so Cd was chosen to represent smoking activity for the multiple linear regression analysis. As expected, the results in Table 7 showed that smoking during sampling can increase indoor Cd concentrations in both seasons. However, it should be noted that outdoor Cd was also an important contributor to indoor Cd. Indoor Cd was correlated with outdoor Cd, *r* = 0.54 in the non-heating season, and 0.41 in the heating season from Spearman analysis. The reason that indoor Cd was sensitive to indoor smoking may due to the relatively low concentration which has already been explained above. 

Apart from the above indoor and outdoor sources, factors related to the exchange of indoor and outdoor air also can affect indoor PM_2.5_. Window ventilation can promote the exchange of indoor and outdoor air to help outdoor PM_2.5_ move into a room [22,56,57,58,59,60,61,62,79]. Natural ventilation was associated with higher indoor PM_2.5_ [79]. In this study, a significant correlation between indoor Pb and time of window open per day was observed in non-heating season, houses with more hours of window opening had higher level of Pb from Table 7. As mentioned before, Pb was chosen as a representative element for outdoor sources, so these results indicate that window ventilation can promote the movement of outdoor PM_2.5_ indoors. However, indoor PM_2.5_ was not correlated with window ventilation, but was inversely related to time of air conditioner use in the non-heating season from Table 7. A possible explanation is that residents tend to close all the windows and reduce indoor and outdoor air exchanges when air conditioners are used to save energy, and air conditioners were also reported to increase indoor PM_2.5_ deposition by a filtering process [80], so indoor PM_2.5_, especially from indoor sources, may decrease when air conditioners are working. No significant correlation between indoor PM_2.5_, Pb and window ventilation in heating season was found in multiple linear regression analysis, which could be partially explained by the short window opening time and small window opening areas, as the median time of window opening per day was only 0.5 h in the heating season. Previous studies suggested that as the building age increases, the airtightness decreases, leading to high infiltration rates of outdoor particles [81,82]. Similar results can also be found in this study. In the heating season, houses with older building age had higher indoor Cd and Pb concentrations.

Meteorological variables may also affect indoor and outdoor air exchanges, thus influencing indoor PM_2.5_ concentrations [22,56,57,58,59,60]. In this study, the outdoor temperature was associated with indoor PM_2.5_ in the non-heating season from Table 7. One possible explanation is that the outdoor temperature ranged from 9.4 to 27.9 °C during sampling in the non-heating season, and residents may tend to increase window ventilation with the increase of temperature, therefore more outdoor PM_2.5_ enters indoors, leading to higher indoor PM_2.5_ concentrations. RH were correlated with indoor Pb in the heating season. High relative humidity often appeared during rainy or snowy weather in the heating season, so residents may close windows and reduce indoor/outdoor air exchange, decreasing the indoor Pb concentration.

Indoor cooking was another identified indoor source of PM_2.5_ [12,22,23,59,83], but a significant correlation between cooking and indoor PM_2.5_ was not observed in our study; this correlation was also not identified in another study conducted in Beijing [61]. One possible explanation is that the cooking time was quite short compared to the entire sampling time, and another study conducted in Beijing suggested that cooking activity can generate sharp but narrow additive pulse peaks of indoor PM_2.5_ concentration [59], so PM_2.5_ may be largely created through cooking, but its contribution to the entire sample may be low. Another explanation is that most houses used a hood and closed the kitchen doors during cooking, and our sampling pumps were usually placed in the living room or bedroom. A previous study also reported that separating the cooking place from the other rooms could reduce indoor PM_2.5_ [84], so, the PM_2.5_ from cooking could hardly be collected. Besides, we did not choose elements representing indoor cooking for multiple linear regression analysis. Though we collected some information related to indoor cooking, such as frequency of cooking, kitchen door and hoods used during sampling, etc., that was not enough because the concentrations of elements from indoor cooking can be greatly influenced by the cooking style, oils and foods used for cooking [41,42], and information about these variables were not collected in this study. Another reason is most of the houses in this study did indoor cooking during the sampling period, so the study lacked a certain number of non-cooking houses as a control to compare and analyze the results. Researchers should take these variables previously mentioned into account for future indoor cooking-related studies. 

Indoor human activities such as movement and cleaning can lead to resuspension of dust and increase the concentration of indoor particles [59,83,85]. A study conducted in Sweden suggested that high concentration of particles mainly occurred during active periods of occupancy, but the variable related to indoor human activities collected in this study was population (number of residents in the house), and no significant correlation was found between population and concentrations of indoor PM_2.5_ and its constituents. Building type also failed to show any significant correlation with indoor PM_2.5_, Cd and Pb. One possible reason is that the residential ventilation behavior in different types of building was basically the same. Another reason is that indoor smoking existed in both courtyards and apartments. Besides Pb and Cd, we also tried to analyze potential influencing factors of other elements from indoor PM_2.5_, and an interesting result was found whereby building type was significantly associated with indoor Tl in the non-heating season, as indoor Tl in courtyards was lower than in apartments and villas. A likely explanation for this result is that Tl may exist in the new building materials which often used for apartments and villas.

However, there are also several limitations in our study. First, there were some potential variables that have not been taken into account in this study, such as indoor cleaning activities and indoor plants, which might affect the indoor PM_2.5_ according to previously published articles [12,22,57,59,61]. Second, houses that use coal and wood for cooking and heating were not included in this study, but the contribution of coal and wood burning to indoor PM_2.5_ and chemical elements is very large according to previous studies [41,69,86]. All the houses in this study did not use fresh air systems and air cleaners during the sampling periods, but they have gradually become widely used in recent years, so their impact on indoor PM_2.5_ should be explored in future studies. Third, the concentrations of PM_2.5_ and its elements in this study were calculated as the average level of the sampling week; therefore, some variables that can influence indoor PM_2.5_ concentrations in the short-term may not be observed and analyzed. Fourth, the data of potential influencing factors mostly were collected by questionnaire and recording sheets, so some information bias may exist in this study. At last, although the number of houses used for analysis in our study was comparable to previous studies, the sample size and the constituent data of indoor PM_2.5_ was still not sufficient, and they should be expanded for wider application of our findings because of the variability of residents’ activity and complexity of indoor PM_2.5_ sources.

## 5. Conclusions

In this study, targeted residential houses were separately and continuously sampled in the non-heating and heating season, and indoor and outdoor concentrations of PM_2.5_ and related elements of urban houses in Beijing were obtained. It was found that indoor concentrations of PM_2.5_ were more than twice higher in smoking houses than in non-smoking houses in the heating season, and indoor PM_2.5_ of smoking houses also higher in the non-heating season, but the difference was not significant. Therefore, people should avoid indoor smoking, particularly in the heating season, to reduce indoor PM_2.5_ concentrations and decrease potential health risks.

It was also found that among elements in indoor PM_2.5_, Cd and As may be sensitive to indoor smoking, Zn, Ca and Al may be related to indoor sources other than smoking, Pb, V and Se may mainly come from outdoors. Five factors were extracted for indoor PM_2.5_, two outdoor sources factors contributed more variance to indoor PM_2.5_, and the contribution of indoor source factor also cannot be ignored. The relationship between indoor concentrations of PM_2.5_ and two selected elements (Cd and Pb) and their potential influencing factors were explored, and it was found that outdoor concentration was an important factor for both PM_2.5_ and chemical elements, indoor smoking can increase indoor PM_2.5_ and Cd concentration, factors including window ventilation, air conditioner use, building age and meteorological variables may be associated with indoor and outdoor air exchange, and then affect indoor concentrations of PM_2.5_ and its constituents.

Several factors influencing indoor concentrations of PM_2.5_ and representative elements were found in this study which should be examined in future studies, and this study can contribute further to accurately assessing indoor PM_2.5_ exposure, and reducing the health impact of indoor PM_2.5_ and its elements on Beijing residents.

## Figures and Tables

**Table 1:** Household characteristics of residential houses in this study.

Household Characteristics	NHS	HS
	*N*	*N*
Number of total houses	47	47
Building type		
apartments	28	26
villas	5	5
courtyards	14	16
Building age		
<10 years	10	6
10–20 years	12	15
>20 years	25	26
Cooking fuel type		
No cooking	3	4
Liquefied gas	12	11
Coal gas	0	4
Nature gas	29	26
Electric	3	2
Presence of indoor cooking	41	40
Presence of indoor smoke	10	10

**Table 2:** Distribution of residential indoor and outdoor concentration of PM_2.5_ and elements (median (IQR: Interquartile Range)).

Constituent	All Seasons (*N* = 94)			NHS (*N* = 47)			HS (*N* = 47)		
	Indoor	Outdoor	*p*_d_^a^	Indoor	Outdoor	*p*_d_^a^	Indoor	Outdoor	*p*_d_^a^
PM_2.5_ (μg/m^3^)	57.64 (27.51)	72.49 (43.53)	<0.01	55.44 (24.12)	65.91 (28.90)	<0.01	58.23 (13.28)	91.85 (26.61)	<0.01
Al (ng/m^3^)	400.34 (274.64)	551.71 (254.69)	<0.01	454.95 (204.34)	540.15 (222.43)	<0.01	306.00 (61.76)	561.01 (94.42)	<0.01
As (ng/m^3^)^a^	9.83 (30.75)	12.16 (31.62)	0.11	6.00 (21.78)	0.16 (12.18)	0.17	18.34 (17.02)	25.81 (16.40)	<0.01
Ca (ng/m^3^)	302.85 (247.21)	433.87 (319.64)	<0.01	288.61 (289.73)	322.95 (132.04)	0.85	322.48 (97.38)	506.56 (68.86)	<0.01
Cd (ng/m^3^)	1.84 (1.19)	1.93 (1.27)	<0.01	1.93 (1.04)	1.86 (1.07)	0.01	1.77 (0.62)	2.11 (0.68)	0.01
Cu (ng/m^3^)	21.28 (12.57)	35.38 (19.21)	<0.01	21.37 (10.54)	24.89 (17.13)	<0.01	20.44 (5.65)	38.15 (6.94)	<0.01
Fe (ng/m^3^)	675.85 (604.24)	1260.48 (997.58)	<0.01	758.80 (582.96)	1260.48 (901.70)	0.05	610.08 (236.62)	1301.99 (486.36)	<0.01
K (ng/m^3^)	930.24 (505.95)	1176.27 (838.15)	<0.01	892.17 (549.59)	1077.51 (632.11)	<0.01	1011.85 (181.24)	1348.57 (247.78)	<0.01
Mg (ng/m^3^)	163.35 (98.45)	225.21 (130.50)	<0.01	163.35 (118.95)	198.89 (123.37)	<0.01	162.82 (41.75)	277.67 (52.46)	<0.01
Mn (ng/m^3^)	36.79 (20.54)	54.44 (28.53)	<0.01	39.07 (23.49)	52.67 (20.38)	<0.01	30.92 (5.87)	71.21 (17.69)	<0.01
Na (ng/m^3^)	439.63 (273.69)	566.85 (274.72)	<0.01	388.72 (290.57)	455.47 (234.50)	0.03	460.08 (91.72)	700.37 (181.91)	<0.01
Pb (ng/m^3^)	105.28 (64.08)	145.01 (81.50)	<0.01	105.66 (52.73)	140.52 (78.05)	<0.01	104.3 (36.06)	162.23 (70.81)	<0.01
Se (ng/m^3^)^a^	3.29 (3.34)	5.35 (3.14)	<0.01	4.15 (3.97)	5.46 (3.34)	<0.01	2.14 (0.75)	5.09 (2.30)	<0.01
Tl (ng/m^3^)	0.92 (0.66)	1.19 (1.17)	<0.01	1.11 (0.65)	1.31 (1.30)	<0.01	0.74 (0.25)	1.11 (0.28)	<0.01
V (ng/m^3^)^a^	1.63 (1.77)	2.40 (2.37)	<0.01	1.71 (1.44)	2.43 (1.51)	<0.01	1.31 (0.80)	2.40 (0.55)	<0.01
Zn (ng/m^3^)	217.1 (133.31)	270.55 (159.02)	<0.01	229.3 (118.85)	270.55 (153.55)	<0.01	207.01 (67.30)	275.63 (62.21)	<0.01

Note: *p*_d_ is the *p*-value of Wilcoxon signed-rank test for comparing indoor and outdoor. There are some houses with concentrations of V, Se and As below the detection limit.

**Table 3:** Comparison of indoor and outdoor concentrations of PM_2.5_ and elements in smoking and non-smoking houses (median).

Constituent	Smoking in NHS (*N* = 10)			Non-Smoking in NHS (*N* = 37)			*p*_NHS_^a^	Smoking in HS (*N* = 10)			Non-Smoking in HS (*N* = 37)			*p*_HS_^a^
	Indoor	Outdoor	*p*_d_	Indoor	Outdoor	*p*_d_		Indoor	Outdoor	*p*_d_	Indoor	Outdoor	*p*_d_	
PM_2.5_ (μg/m^3^)	66.92	59.01	0.96	53.51	70.62	<0.01	0.13	129.34	72.04	0.33	54.60	91.85	<0.01	<0.01
Al (ng/m^3^)	479.09	731.31	0.11	444.88	532.21	<0.01	0.86	317.14	551.53	0.28	306.00	561.01	<0.01	0.78
As (ng/m^3^)	17.05	0.13	0.02	2.97	1.21	0.99	0.21	32.58	31.37	0.11	16.37	22.37	0.01	0.29
Ca (ng/m^3^)	459.64	284.33	0.11	272.28	370.99	0.34	0.15	220.36	459.35	0.07	335.5	630.97	<0.01	0.06
Cd (ng/m^3^)	2.64	1.34	0.01	1.80	2.10	<0.01	0.02	3.93	1.55	0.01	1.34	2.20	<0.01	<0.01
Cu (ng/m^3^)	18.61	23.06	0.17	22.76	30.31	<0.01	0.12	25.26	38.98	0.11	20.44	38.15	<0.01	0.32
Fe (ng/m^3^)	990.32	1578.39	0.28	726.54	905.30	0.11	0.34	673.13	785.05	0.96	575.63	1851.86	<0.01	0.47
K (ng/m^3^)	923.04	846.53	0.14	892.17	1157.92	<0.01	0.36	1277.48	1193.32	0.88	962.13	1391.27	<0.01	0.07
Mg (ng/m^3^)	190.01	151.40	0.51	160.41	201.22	<0.01	0.92	149.72	215.95	0.04	170.68	279.87	<0.01	0.17
Mn (ng/m^3^)	40.38	48.69	0.20	39.07	53.26	<0.01	0.9	28.66	42.60	0.20	31.09	71.61	<0.01	0.79
Na (ng/m^3^)	428.54	376.37	0.20	388.72	472.35	<0.01	0.47	492.05	593.78	0.96	455.51	700.67	<0.01	0.22
Pb (ng/m^3^)	112.39	114.91	0.33	105.66	140.52	<0.01	0.70	85.17	98.72	0.01	107.92	163.95	<0.01	0.18
Se (ng/m^3^)	5.21	4.21	0.88	3.86	5.54	<0.01	0.11	1.44	3.91	0.02	2.48	5.09	<0.01	0.36
Tl (ng/m^3^)	1.15	1.12	0.17	1.11	1.31	<0.01	0.79	0.74	0.88	0.11	0.72	1.16	<0.01	0.90
V (ng/m^3^)	2.10	2.43	0.01	1.71	2.43	<0.01	0.66	1.80	2.09	0.44	1.22	2.40	<0.01	0.21
Zn (ng/m^3^)	254.30	189.75	0.39	229.30	271.48	<0.01	0.47	144.57	185.22	0.06	218.21	333.03	0.01	0.07

Note: *p*_NHS_ is the *p*-value of Mann-Whitney U test for comparing smoking and non-smoking houses in non-heating season, *p*_HS_ is the *p*-value for comparing smoking and non-smoking houses in heating season.

**Table 4:** Comparison of indoor and outdoor concentrations of PM_2.5_ and elements for houses with window close in HS (median).

Constituent	Windows Close (*N* = 19)			Windows Close and Non-Smoking (*N* = 14)		
	Indoor	Outdoor	*p*_d_	Indoor	Outdoor	*p*_d_
PM_2.5_ (μg/m^3^)	69.53	84.27	0.40	61.77	84.27	0.04
Al (ng/m^3^)	312.72	517.09	0.09	306	482.46	0.29
As (ng/m^3^)	7.89	18.78	0.14	6.16	14.8	0.04
Ca (ng/m^3^)	226.63	517.79	0.17	241.69	624.56	0.35
Cd (ng/m^3^)	1.28	1.92	0.18	1.20	1.92	<0.01
Cu (ng/m^3^)	18.45	38.15	<0.01	16.56	36.86	<0.01
Fe (ng/m^3^)	622.17	1851.86	0.02	610.08	1988.55	<0.01
K (ng/m^3^)	928.34	1320.13	0.01	896.54	1255	<0.01
Mg (ng/m^3^)	130.37	275.89	0.01	122.18	275.89	0.02
Mn (ng/m^3^)	31.55	71.21	<0.01	29.19	72.78	<0.01
Na (ng/m^3^)	428.01	669.73	0.06	369.75	700.37	<0.01
Pb (ng/m^3^)	105.32	163.95	<0.01	102.75	163.09	<0.01
Se (ng/m^3^)	1.47	4.33	0.01	1.46	3.96	0.04
Tl (ng/m^3^)	0.69	1.04	<0.01	0.52	1.02	<0.01
V (ng/m^3^)	2.18	5.04	<0.01	2.04	5.16	<0.01
Zn (ng/m^3^)	175.14	246.72	0.16	183.91	299.4	0.48

**Table 5:** Comparison of indoor and outdoor concentrations of PM_2.5_ and elements for houses that sampled in both seasons (median, *N* = 37).

Constituent	NHS			HS			*p*_in_^a^	*p*_out_^a^
	Indoor	Outdoor	*p*_d_	Indoor	Outdoor	*p*_d_		
PM_2.5_ (μg/m^3^)	53.51	70.62	<0.01	54.54	91.85	<0.01	0.71	0.02
Al (ng/m^3^)	457.83	609.78	<0.01	299.88	561.01	<0.01	0.36	0.51
As (ng/m^3^)	2.99	0.16	0.41	26.07	31.37	0.01	0.04	<0.01
Ca (ng/m^3^)	288.61	370.99	0.51	319.11	591.58	<0.01	0.84	<0.01
Cd (ng/m^3^)	1.93	1.86	0.03	1.82	2.11	<0.01	0.56	0.82
Cu (ng/m^3^)	22.56	29.50	<0.01	19.47	38.15	<0.01	0.32	<0.01
Fe (ng/m^3^)	749.38	1134.84	0.04	610.08	1132.16	<0.01	0.09	0.12
K (ng/m^3^)	911.35	1157.92	<0.01	976.56	1391.27	<0.01	0.51	0.01
Mg (ng/m^3^)	166.34	201.22	<0.01	162.82	279.87	<0.01	0.57	<0.01
Mn (ng/m^3^)	40.90	52.67	<0.01	29.19	71.21	<0.01	0.04	0.03
Na (ng/m^3^)	398.56	511.57	0.01	460.08	700.37	<0.01	0.44	<0.01
Pb (ng/m^3^)	105.66	140.52	<0.01	93.16	147	<0.01	0.46	0.10
Se (ng/m^3^)	4.15	5.46	<0.01	2.48	5.09	<0.01	<0.01	0.20
Tl (ng/m^3^)	1.16	1.36	<0.01	0.74	1.11	<0.01	<0.01	0.21
V (ng/m^3^)	1.71	2.43	<0.01	0.77	2.15	<0.01	<0.01	0.24
Zn (ng/m^3^)	229.3	271.48	<0.01	218.21	275.12	<0.01	0.38	0.68

Note: *p*_in_ is the *p*-value of Wilcoxon signed-rank test for comparing indoor concentration of the same houses in two seasons. *p*_out_ is the *p*-value for comparing outdoor concentration of the same houses in two seasons.

**Table 6:** Factor analysis for indoor PM_2.5_ (all seasons).

Elements	F1	F2	F3	F4	F5	Communality
Al				0.71		0.6
As					0.83	0.83
Ca			0.92			0.86
Cd	0.72				0.56	0.88
Cu	0.60	0.53				0.66
Fe	0.89					0.86
K		0.72				0.82
Mg			0.54	0.61		0.74
Mn	0.74	0.45				0.90
Na				0.74		0.73
Pb		0.78				0.72
Se		0.78				0.71
Tl		0.82				0.68
V	0.78					0.75
Zn			0.86			0.78
Eigenvalue	5.02	2.32	1.98	1.12	1.07	
% of variance	21.55	21.28	13.80	11.37	8.79	
Cumulative %	21.55	42.82	56.62	68.00	76.78	
Possible source type	Combustion	Motor vehicles and combustion	Indoor cooking	Dust and soil	Indoor smoking	

**Table 7:** Multiple linear regression of indoor PM_2.5_ and selected elements and their potential influencing variables.

Variable	Coefficient ^a^			*t* ^a^	*p* ^a^
	β ^a^	SE ^a^	β’ ^a^		
PM_2.5_ in NHS (R^2^ = 0.60)					
Intercept	1.106	0.122		9.102	<0.001
Outdoor PM_2.5_	0.006	0.001	0.868	6.296	<0.001
Smoking during sampling	0.128	0.034	0.386	3.726	0.001
Outdoor temperature	0.010	0.003	0.440	3.057	0.004
Time of air conditioner use during sampling	−0.002	0.001	−0.269	−2.251	0.030
PM_2.5_ in HS (R^2^ = 0.46)					
Intercept	1.482	0.065	-	22.712	<0.001
Outdoor PM_2.5_	0.003	0.001	0.462	4.167	<0.001
Smoking during sampling	0.266	0.063	0.468	4.225	<0.001
Cd in NHS (R^2^ = 0.51)					
Intercept	−0.264	0.087	-	−3.047	0.004
Smoking during sampling	0.350	0.066	0.603	5.344	<0.001
Outdoor Cd	0.205	0.036	0.642	5.692	<0.001
Cd in HS (R^2^ = 0.63)					
Intercept	−1.228	0.225	-	−5.446	<0.001
Smoking during sampling	0.713	0.120	0.562	5.957	<0.001
Outdoor Cd	0.321	0.050	0.605	6.379	<0.001
Building age	0.219	0.070	0.298	3.145	0.003
Pb in NHS (R^2^ = 0.63)					
Intercept	1.421	0.069		20.673	<0.001
Outdoor Pb	0.004	0.001	0.739	8.003	<0.001
Time of window open per day	0.006	0.002	0.247	2.681	0.010
Pb in HS (R^2^ = 0.74)					
Intercept	1.357	0.124	-	10.973	<0.001
Outdoor Pb	0.004	0.001	1.009	9.252	<0.001
Building age	0.099	0.030	0.260	3.320	0.002
RH	−0.008	0.004	−0.232	−2.143	0.038

Note: β is the partial regression coefficient for each independent variable, SE is the standard error of β, β’ is the standard regression coefficient, *t* is the *t*-value of the regression coefficient significance test (*t*-test) and *P* is the *p*-value of *t*-test.

## Supplementary Materials

The following are available online at http://www.mdpi.com/1660-4601/15/4/686/s1, Table S1: Main summary of the baseline questionnaire and sampling questionnaire, Table S2: Summary of detection limits for chemical elements in this study.
