# Supplementary material for: Source Apportionment and Influencing Factor Analysis of Residential Indoor PM2.5 in Beijing
Source: Int J Environ Res Public Health. 2018 Apr 5;15(4):686. doi: 10.3390/ijerph15040686 (PMC5923728; doi:10.3390/ijerph15040686)
Supplement: Supplementary file 1 [file ijerph-15-00686-s001.pdf]

Supplementary Materials:

Table S1. Main summary of the baseline questionnaire and sampling questionnaire.

Variables	Questions	Response(s)
Baseline questionnaire		
household characteristics		
building type	What type of building is your house?	apartment, villa, courtyard
building age	How old is your house?	specific years
building height	What floor is your house on?	specific floors
room number	How many rooms are in your house?	specific room number
total household income(Yuan)	Normally, what annual income range does your family belong to?	<50000,50000-100000,100000-150000,>150000
population	Normally, how many people live in your house?	specific population number
Indoor source		
number of smokers	Normally, how many smokers live in your house?	specific smoker number
average frequency of cooking each day	Normally, what is the average frequency of cooking each day in your house?	0,1,2,3,other
average time to cook a meal(min)	Normally, what is the average time to cook a meal in your house?	<20,20-40,40-60,>60mi
cooking fuel type	Normally, what type of cooking fuel do you use in your house?	liquefied petroleum gas, coal gas, nature gas, electric
hoods	Normally, do you use a hood during cooking in your house?	Yes or No
Heating and ventilation		
air conditioner(AC) use	Normally, do you use an AC in your house?	Yes or No
heating type	What type of heating does your house use?	centralized heating, AC, electric heating, other
window type	What type of windows does your house have?	uphill extrapolation, forward and backward extrapolation, up and down pull, left and right pull
number of open windows	Normally, how many windows do you open every day in the non-heating/heating period?	all, some, none
open window width	Normally, what is the percentage of windows you open in the	<10%,10-20%,20-50%,50-80%,100%

	non-heating/heating period?	
open window time	Normally, for how many hours do you open windows each day during the non-heating/heating period?	specific hours
air cleaner use	Normally, do you use air cleaner in your house?	Yes or No
Sampling questionnaire		
Indoor source		
smoking during sampling	Was there anyone (include guests) smoking in your house during sampling?	Yes or No
number of smokers per day	How many smokers smoked in your house per day during sampling?	specific smoker number
number of cigarettes per day	What was the number of cigarettes smoked per day in your house during sampling?	specific number of cigarettes
average frequency of cooking per day	What was the average frequency of cooking per day in your house during sampling?	0,1,2,3,other
average time to cook a meal per day(min)	What was the average time to cook a meal per day in your house during sampling?	<20,20-40,40-60,>60
hoods used during sampling	What was the frequency of hood use in your house during sampling?	All the cooking time, half the cooking time, hardly use hood
Heating and ventilation		
AC use during sampling	Did you use an AC in your house during sampling?	Yes or No
temperature of AC	What was the temperature setting for the AC?	specific temperature
frequency of AC use	What was the frequency of AC use in your house during sampling?	Almost daily ,more than half the days of sampling, less than half the days of sampling, hardly use AC
heating use	Did you use heating in your house during sampling?	Yes or No
number of windows open per day	How many windows did you open per day during sampling?	all, some, none
width of window open per day	What was the percentage of windows open per day during sampling?	<10%,10-20%,20-50%,50-80%,100%
time of window open per day	What was the average window open time per day in your house	specific hours

	during sampling?	
fresh air system use	Do you use fresh air system in your house during sampling?	Yes or No
air cleaner use	Do you use air cleaner in your house during sampling?	Yes or No

Table S2. Summary of operation conditions of the ICP-MS in this study.

Instrument parameter	Setting
Spray chamber	Cyclonic
Nebulizer gas flow	0.92 L/min
Cones	Pt
Auxiliary gas flow	1.20 L/min
Plasma gas flow	18 L/min
ICP RF power	1400 W
Scan mode	Peak hoping
Detector mode	Dual
Analog stage voltage	−1725
Pulse stage voltage	800
Discriminator threshold	11
Quadrupole rod offset	0
Scan mode	Peak hoping
Detector mode	Dual
Process spectral peak	Average
Process signal profile	Average
Dwell time	100 ms
Sweeps/reading	20
Replicates	3
Sweeps/reading	20
Replicates	3

Table S3. Summary of detection limits for chemical elements in this study.

Elements	Detection Limits
Al(ug/L)	4.0
V(ng/L)	58.7
Mn(ug/L)	0.2
Fe(ug/L)	31.7
Cu(ug/L)	0.4
Zn(ug/L)	2.3
As(ng/L)	53.4
Se(ug/L)	0.6
Cd(ng/L)	5.0
Tl(ng/L)	0.8
Pb(ng/L)	59.5
Na(ug/L)	6.4
Mg(ug/L)	4.8
K(ug/L)	9.6
Ca(ug/L)	19.0
